# Development of a new set of molecular markers for examining *Glu-A1* variants in common wheat and ancestral species

**DOI:** 10.1371/journal.pone.0180766

**Published:** 2017-07-06

**Authors:** Zhenying Dong, Yushuang Yang, Kunpu Zhang, Yiwen Li, Junjun Wang, Zhaojun Wang, Xin Liu, Huanju Qin, Daowen Wang

**Affiliations:** 1 The State Key Laboratory of Plant Cell and Chromosomal Engineering, Institute of Genetics and Developmental Biology, Chinese Academy of Sciences, Beijing, China; 2 Rubber Research Institute, Chinese Academy of Tropical Agricultural Sciences, Danzhou, China; 3 Graduate University of Chinese Academy of Sciences, Beijing, China; 4 The Collaborative Innovation Center for Grain Crops, Henan Agricultural University, Zhengzhou, China; USDA, UNITED STATES

## Abstract

In common wheat (*Triticum aestivum* L.), allelic variations of *Glu-A1* locus have important influences on grain end-use quality. Among the three *Glu-A1* alleles, *Glu-A1a* and -*A1b* encode the high-molecular-weight glutenin subunits (HMW-GSs) 1Ax1 and 1Ax2*, respectively, whereas *Glu-A1c* does not specify any subunit. Here, we detected a total of 11 *Glu-A1* locus haplotypes (H1 to H11) in three wheat species, by developing and using a new set of DNA markers (*Xrj5*, *Xid3*, *Xrj6*, *Xid4* and *Xrj7*). The main haplotypes found in the diploid wheat *T*. *urartu* were H1, H4, H5 and H6, with H1 and H4 expressing both 1Ax and 1Ay subunits. The major haplotypes revealed for tetraploid wheat (*T*. *turgidum*) were H1, H8 and H9, with the lines expressing both 1Ax and 1Ay belonging to H1, H4 or H7. Four major haplotypes (H1, H9, H10 and H11) were discovered in common wheat, with *Glu-A1a* associated with H1 and H8, *Glu-A1b* with H10 or H11, and *Glu-A1c* with H9. The *Glu-A1* locus haplotypes and the new set of DNA markers have potential to be used for more effectively studying and utilizing the molecular variations of *Glu-A1* to improve the end-use quality of common wheat are discussed.

## Introduction

Common wheat (*Triticum aestivum*, 2n = 42, AABBDD) is one of the most important staple food crops in the world, providing 20% of dietary energy and protein sources for over 60% of the world population [1, 2]. *T*. *aestivum* was originated about 10,000 years ago due to natural hybridization between tetraploid wheat (*T*. *turgidum*, 2n = 28, AABB) and the D genome donor *Aegilops tauschii* (2n = 14, DD) [3, 4]. *T*. *turgidum* was derived from the hybridization between *T*. *urartu* (2n = 14, AA) and an unknown species of the *Sitopsis* section about 0.5 million years ago [5]. Both *T*. *urartu* and *T*. *turgidum* contain the A genome, which is closely related to the A genome present in bread wheat.

Compared with other major cereal crops (such as rice and maize), wheat is unique in that its flour can be processed into multiple types of food products (e.g., bread, noodles and cakes). This is owing to the formation of wheat doughs with different viscoelastic properties [6]. It is now well known that wheat dough properties are mainly determined by three families of gluten proteins, high-molecular-weight glutenin subunits (HMW-GSs), low-molecular-weight glutenin subunits (LMW-GSs) and gliadins, present in the flour [6, 7]. HMW-GSs are the major determinant of dough elasticity, whereas LMW-GSs tend to contribute to both dough elasticity and extensibility [7, 8]. Gliadins may contribute to dough extensibility [9]. In common wheat, the genes encoding HMW-GSs are contained in three homoeologous *Glu-1* loci (*Glu-A1*, -*B1* and -*D1*) located on homoeologous group 1 chromosomes [8]. In each locus, there are two duplicated HMW-GS genes, designating as x- and y-type subunits, respectively. The HMW-GS proteins that have been characterized to date all share a conserved primary structure composed of a signal peptide (removed from mature subunit), a N-terminal domain, a central repetitive domain, and a C-terminal domain [8]. The N-, C- and central repetitive domains all contribute to the function of HMW-GSs in end-use quality control [8, 10].

Within each *Glu-1* locus, the two HMW-GS genes are usually separated by a large distance (54–190 kb), with multiple additional genes and transposable elements (TEs) distributed in the vicinity of x- and y-type genes [11–13]. Comparisons of allelic *Glu-1* loci have revealed nucleotide sequence variations in not only HMW-GS genes but also the surrounding genes and TEs [11–13]. However, at present, only nucleotide sequence variations in HMW-GS genes are considered in differentiating different *Glu-1* alleles. These variations frequently lead to the expression of allelic x- and y-type subunits differing in electrophoretic mobility on SDS-PAGE, or the silencing of one or more HMW-GS genes in certain genotypes. Consequently, *Glu-A1*, *-B1* and *-D1* have all been found to have multiple alleles, and *Glu-B1* has the most alleles, followed by *Glu-D1* and *Glu-A1* [14]. Importantly, *Glu-1* alleles have been found to differ considerably in their effects on dough functionality and end-use quality. For example, in *Glu-D1*, the *Glu-D1d* allele (encoding 1Dx5 and 1Dy10 subunits) is functionally superior to *Glu-D1a* (specifying 1Dx2 and 1Dy12) [15–17].

To date, three *Glu-A1* alleles have been defined in bread wheat; *Glu-A1a* (expressing 1Ax1 subunit) and *Glu-A1b* (Specifying 1Ax2*) alleles have positive effects on breadmaking quality whereas *Glu-A1c* (not expressing any HMW-GS because of gene silencing) has a low quality score [9, 15]. One common feature shared by the three alleles is that the gene encoding y-type subunit is silenced [11, 18–20]. Interestingly, this gene is active in some *T*. *urartu* and *T*. *turgidum* accessions [21–26]. However, in these species, the *Glu-A1* genomic region is still poorly understood, and the *1Ax* and *1Ay* genes in this species are largely uncharacterized at the molecular level. Moreover, the allelic differentiation of *Glu-A1* in *T*. *urartu* and *T*. *turgidum* populations has not been systematically studied using molecular markers, although SDS-PAGE data have shown that the expression of *1Ax* and *1Ay* genes varied extensively within and between the two species [24, 26].

Because allelic variation of *Glu-1* loci affects their function in end-use quality control, substantial efforts have been devoted to mine functionally superior *Glu-1* alleles in wheat and related species [27–30]. In a previous study, we found that DNA marker-mediated haplotype analysis was highly efficient for revealing the molecular variations of *Glu-D1* genomic region, tracing the origin of the superior *Glu-D1d* allele, and guiding the mining of more *Glu-D1d* variants in specific wheat species [31]. Therefore, the major objectives of this study were to develop a new set of DNA markers for *Glu-A1* genomic region and to use them for investigating the haplotype variants of *Glu-A1* locus. The relationships between *Glu-A1* locus haplotype variants and the expression of 1Ax and/or 1Ay subunits were also explored. Our data should facilitate more effective study and utilization of *Glu-A1* variants in improving the end-use quality of common wheat in further research.

## Materials and methods

### Plant materials

A total of 99 *T*. *urartu* accessions (S1 Table), 95 *T*. *turgidum* lines (S2 Table) and 215 common wheat varieties (S3 Table) were employed in this study. For validating the specificity of newly developed *Glu-A1* DNA markers, the nulli-tetrasomic line N1AT1D and the ditelosomic line Dt1AS of Chinese spring (CS) [32, 33] were applied.

### Marker development and haplotype analysis

The BAC sequences of DQ537335 (from common wheat variety Renan, *T*. *aestivum*), AY494981 (from durum wheat variety Langdon, *T*. *turgidum*) and JQ240472 (from *T*. *urartu* accession G1812), each carrying a completely sequenced *Glu-A1* locus, were retrieved from the NCBI database (http://www.ncbi.nlm.nih.gov/). TE insertions and deletions and short nucleotide indels in the three sequences were considered for marker development. The software primer premier 5.0 (PREMIER Biosoft International, USA) was used for designing the primer pairs specific for each of the five *Glu-A1* markers (S4 Table). To verify the A genome specificity of the designed primers, the BAC sequences from Renan *Glu-B1* (DQ537336) and *Glu-D1* (DQ537337) loci were also used for sequence alignment. Genomic DNA samples were extracted from desired plant lines grown in the greenhouse using a cetyltrimethyl ammonium bromide (CTAB) method [34], and used for PCR amplifications. *Glu-A1* locus haplotypes were based on differences in the alleles of the five diagnostic markers.

### PCR and electrophoresis conditions for five *Glu-A1* markers

PCR was carried out in 20 μl volume containing 50 ng genomic DNA template, 10 mM dNTPs, 5 pmol of each primer, and 1 U Taq polymerase (Transgen Biotech, China). The cycling parameters were 94°C for 5 min, followed by 35 cycles of 94°C for 30 s, 60°C for 30s and 72°C for 1 min, and a final extension at 72°C for 5 min. PCR products were separated in 1.5% agarose gels.

### SDS-PAGE analysis of HMW-GSs

HMW-GSs were extracted from the desired grain samples, and separated in 10% SDS-PAGE as detailed previously [35]. HMW-GSs expressed in the winter wheat variety Bobwhite (1Ax2*, 1Bx7, 1By9, 1Dx5 and 1Dy10) [36], Xiaoyan 54 (1Ax1, 1Bx14, 1By15, 1Dx2 and 1Dy12) [37], and Jimai 20 (1Ax1, 1Bx13, 1By16, 1Dx4 and 1Dy12) [38] were used as reference in determination of HMW-GS composition in *T*. *urartu*, *T*. *turgidum* and *T*. *aestivum* lines.

### Cloning the nucleotide sequences of the *1Ay* gene of *T*. *urartu* accession PI428339

The primers and PCR conditions described by Bai et al. 2004 [39] were used to amplify the complete coding sequence of *1Ay*. The target PCR fragment was isolated from agarose gel, and then cloned into the pGEM-T Easy vector (Promega, USA). Three independent clones were sequenced. For multiple sequence alignment among the *1Ay* cloned here and those published previously, two *1Ay* pseudogenes (GenBank accessions AY245579 and EU984505) and five *1Ay* active genes (AY245578, EU984503, EU984504, FJ404595 and JQ240472) isolated from different *T*. *urartu* materials were retrieved from the NCBI database (https://www.ncbi.nlm.nih.gov/).

### Phylogenetic network analysis

The alleles of the five *Glu-A1* markers were employed for phylogenetic network analysis of *Glu-A1* locus haplotypes using the software network 4.6.1.3 (Fluxus Technology Ltd., UK).

## Results

### Development of a new set of DNA markers for *Glu-A1* locus

**Fig 1:**
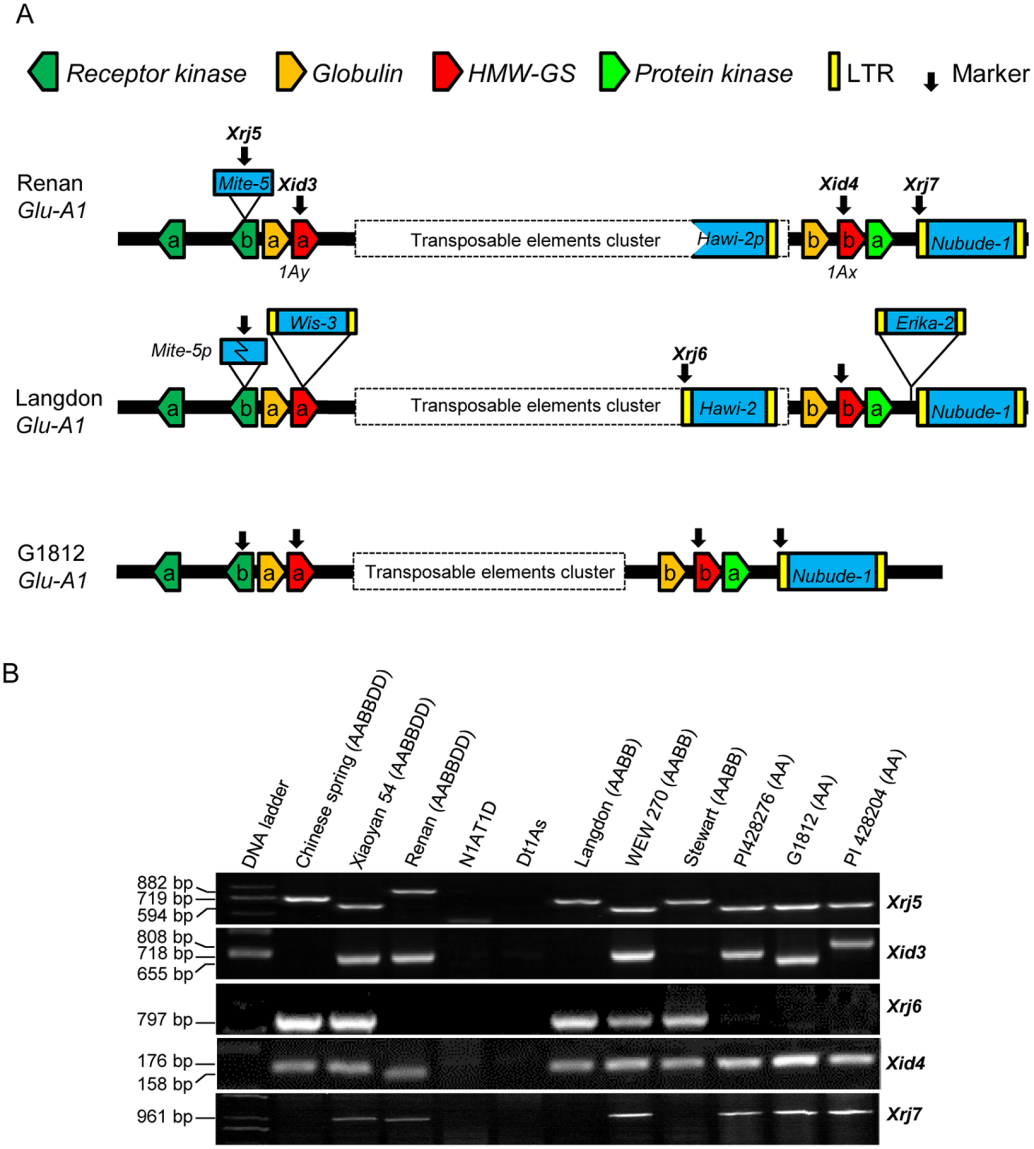
Development of five new molecular markers in *Glu-A1* region. (A) Organization of homologous *Glu-A1* regions in the common wheat variety Renan, durum wheat Langdon and *T*. *urartu* G1812. The diagrams illustrating the organization of the various transposon elements and genes in the three *Glu-A1* regions were adapted from previously published works [11–13]. Only the structures relevant to this work are shown. The letters ‘‘a” and ‘‘b” denote distinct copies of the duplicated genes. Positions of the five newly developed DNA markers (*Xid3*, *Xid4*, *Xrj5*, *Xrj6* and *Xrj7*) are indicated by arrows. (B) Validation of the chromosomal specificity of the five newly developed *Glu-A1* markers. The names and genome constitution of the materials were indicated on the top of the graph. The names of five markers were indicated on the right of corresponding photo, the corresponding length of PCR amplification product was indicated on the left.

The genomic sequences, available for *Glu-A1* regions in the *T*. *urartu* accession G1812, the durum wheat variety Langdon and the common wheat variety Renan [11–13], permitted a detailed comparison of the structural variations of this locus among A genomes from three species. The *Glu-A1* allele of Renan encoded 1Ax2* [40] and thus belonged to *Glu-A1b*; the *Glu-A1* allele of Langdon was *Glu-A1c* that encodes a null allele; the *Glu-A1* allele of G1812 expressed both 1Ax and 1Ay proteins, and thus differed from the three *Glu-A1* alleles defined previously [9, 15]. The patterns of TE insertion and deletion varied considerably among the three *Glu-A1* regions [11–13]. These variations permitted us to develop a new set of DNA markers for investigating the haplotype variants of *Glu-A1* locus (Fig 1A).

*Xid3* and *Xid4* were two indel markers. *Xid3* was developed based on the polymorphism in the coding sequences of the three *1Ay* genes. A 63 bp indel was found between the *1Ay* coding regions of Renan and G1812 (S1 Fig), while a LTR retrotransposon (*Wis-3*, 8633 bp) was observed in the *1Ay* open reading frame (ORF) of Langdon (Fig 1A). *Xid4* was developed based on a 18 bp deletion in the *1Ax* ORF of Renan (S2 Fig, Fig 1A). This deletion did not occur in the *1Ax* gene of G1812 and Langdon. Moreover, the forward and reverse primers used for amplifying *Xid3* or *Xid4* did not have identical binding sites in either *Glu-B1* or *Glu-D1* regions (S1 and S2 Figs), indicating *Xid3* and *Xid4* should be *Glu-A1* specific.

*Xrj5*, *Xrj6* and *Xrj7* were three repeat DNA insertion site based polymorphism (ISBP) markers [41, 42]. *Xrj5* was developed to diagnose the presence of an intact miniature inverted repeat transposable element (*Mite-5*) in the ORF of the receptor kinase b gene in Renan, which was truncated in Langdon and absent in G1812 (Fig 1A, S3 Fig). *Xrj6* resided between *1Ay* and *1Ax* genes, and was designed based on the polymorphisms exhibited by the retroelement *Hawi-2*; this element was relatively intact in Langdon, but was partially deleted (resulting in *Hawi-2p*) in Renan and completely absent in G1812 (Fig 1A). *Xrj7* was located downstream of *1Ax*, and designed based on another retrotransposon *Nobude-1* (Fig 1A). This marker should not amplify PCR products from Langdon owing to the insertion of *Erika-2* (13651 bp) upstream of *Nobude-1* (Fig 1A). *Mite-5*, *Hawi-2* and *Nobude-1* were absent in the *Glu-B1* or *Glu-D1* regions, indicating that the three ISBP markers were likely *Glu-A1* specific.

The A genome specificity and ability of the five developed markers to reveal potential haplotype variations of *Glu-A1* region was tested using three common wheat varieties carrying *Glu-A1a* (Xiaoyan 54), -*A1b* (Renan) and *-A1c* (Chinese spring) alleles, respectively. In addition, three tetraploid wheat lines (Langdon, Stewart and WEW270) and three *T*. *urartu* accessions (PI428281, G1812 and PI428204) were also included in the test. From Fig 1B, it is clear that the amplicons of the five markers were all polymorphic. No products were amplified in the line lacking chromosome 1A (N1AT1D) or 1AL (Dt1AS) (Fig 1B), which validated the location and specificity of the five markers on 1AL chromosome arm.

*Xrj5* yielded three types of amplicons, i.e., 882 bp from Renan, 719 bp from Langdon and 594 bp from G1812 (Fig 1B, S3 Fig). The 719 bp amplicon was also amplified in Chinese Spring, and the 594 bp fragment was also detected in Xiaoyan 54 and WEW 270 (Fig 1B). *Xid3* amplified a null allele in Langdon, a 718 bp fragment in Renan, and a 665 bp fragment in G1812; the null allele was also found in Chinese Spring and Stewart, but an unexpected allele of 808 bp was detected in PI428204 (Fig 1B). This 808 allele was caused by an insertion in the *1Ay* gene of PI428204 (S1 Fig). *Xrj6* amplified the expected fragment (797 bp) in Langdon and a null allele in Renan and G1812; the 797 bp fragment was also detected in Chinese Spring, Xiaoyan 54, Stewart and WEW270 (Fig 1B). The amplicon of *Xid4* was either 158 or 176 bp due to a 18 bp deletion in the *1Ax* gene of Renan (Fig 1B, S2 Fig). Lastly, *Xrj7* amplified the anticipated fragment (961 bp) in Renan and G1812 and a null allele in Langdon, with the 961 bp fragment being also amplified in Xiaoyan 54, WEW270 and two other *T*. *urartu* accessions (Fig 1B). Together, these results suggested that the five markers could reveal the polymorphisms existed in *Glu-A1* genomic region in diploid, tetraploid and hexaploid wheat species, and were thus useful for analyzing the haplotype variations of *Glu-A1* locus.

### Analysis of *Glu-A1* locus haplotypes in *T*. *urartu*

Ninety-nine *T*. *urartu* accessions from Lebanon and Turkey were genotyped and seven *Glu-A1* locus haplotypes were identified. These haplotypes were designated as H1 to H7, respectively (Table 1, S1 Table). In the examined *T*. *urartu* lines, both *Xrj5* and *Xrj7* were monomorphic (with the amplicon sizes of 594 and 961 bp, respectively), whereas *Xid3* derived from *1Ay* gene (Fig 1A) showed the highest level of polymorphism with three different amplicons (Table 1). *Xrj6* and *Xid4* each yielded two alleles (Table 1).

**Table 1:** *Glu-A1* locus haplotypes detected in *T*. *urartu*, *T*. *turgidum* and common wheat populations.

Haplotype	Marker					Species
	*Xrj5*	*Xid3*	*Xrj6*	*Xid4*	*Xrj7*	
**H1**	594^a^	718	797	176	961	*T*. *urartu* (30, 30.30%)^c^ *T*. *turgidum* (37, 38.95%) *T*. *aestivum* (117, 54.42%)
**H2**	594	808	797	176	961	*T*. *urartu* (2, 2.02%)
**H3**	594	655	797	176	961	*T*. *urartu* (1, 1.01%)
**H4**	594	718	-^b^	176	961	*T*. *urartu* (27, 27.27%) *T*. *turgidum* (1, 1.01%)
**H5**	594	808	-	176	961	*T*. *urartu* (20, 20.20%)
**H6**	594	655	-	176	961	*T*. *urartu* (18, 18.18%)
**H7**	594	718	-	158	961	*T*. *urartu* (1, 1.01%) *T*. *turgidum* (1, 1.01%)
**H8**	719	718	797	176	961	*T*. *turgidum* (9, 9.47%) *T*. *aestivum* (1, 0.47%)
**H9**	719	-	797	176	-	*T*. *turgidum* (46, 48.42%) *T*. *aestivum* (79, 36.74%)
**H10**	719	718	-	158	961	*T*. *aestivum* (13, 6.28%)
**H11**	882	718	-	158	961	*T*. *aestivum* (5, 2.33%)

^a^Length (bp) of amplicon by the corresponding marker.

^b^Null allele.

^c^Values in the brackets indicate the number and percentage of lines in which the given *Glu-A1* haplotype was detected.

Among the seven haplotypes found in *T*. *urartu*, H1 was the most common that was found in 30% of the lines, followed by H4 (detected in 27% of the lines). The only difference between H1 and H4 was the presence of *Xrj6* but absence in H4 (Table 1). The amplicon size of *Xid4* was 158 bp in H7 but 176 bp in H1 to H6 (Table 1). Regarding the three amplicons of *Xid3*, the 718 bp fragment was shared by H1, H4 and H7, the 808 bp fragment by H2 and H5, and the 655 bp fragment by H3 and H6 (Table 1). H1, H4, H5 and H6 were considered as major haplotypes because each was found in more than 15 different *T*. *urartu* accessions (Table 1).

**Fig 2:**
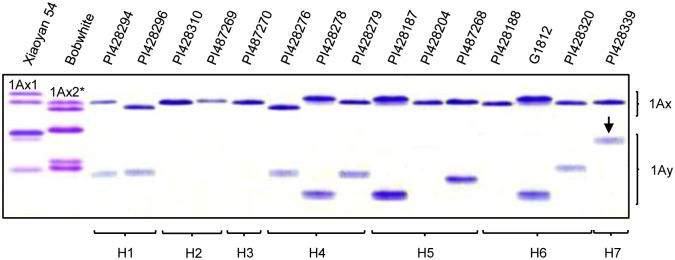
SDS-PAGE detection of HMW-GS composition in *T*. *urartu Glu-A1* haplotypes. The names of materials detected were marked on the top of the photo. The protein bands represented the *T*. *urartu* 1Ax or 1Ay subunits were marked on the right. The corresponding haplotypes were marked on the bottom. The arrow indicated the 1Ay-428339 that displayed the slowest electrophoretic mobility compared with other *T*. *urartu* 1Ay subunits.

HMW-GS composition was analyzed in the 99 *T*. *urartu* accessions carrying different *Glu-A1* locus haplotypes using SDS-PAGE (Fig 2). 1Ax protein was expressed in all 99 lines, whereas 1Ay was detected in 77 lines (S1 Table). Both 1Ax and 1Ay exhibited variations in electrophoretic mobility, with the magnitude of the variations being considerably larger for 1Ay (Fig 2). Among the four major haplotypes (H1, H4, H5 and H6), H1 and H4 contained the accessions that expressed both 1Ax and 1Ay, with 1Ay silenced most frequently in the accessions of H5 (S1 Table).

Interestingly, the 1Ay protein detected in the accession PI428339 (tentatively designated as 1Ay-PI428339) displayed the slowest electrophoretic mobility (Fig 2, arrowed). We thus cloned the coding region of *1Ay-PI428339* (GenBank accession number MF098417) by PCR, and found that the amplicon was 1899 bp. Analysis of translated amino acid sequence showed that 1Ay-428339 contained a signal peptide of 21 amino acids (aa), a conserved N-terminal region, a central repetitive domain and a C-terminal region (S4 Fig). But the length of the C-terminal region of 1Ay-428339 (20 aa) was much shorter than that of typical 1Ay C-terminal region (42 aa) (S4 Fig). A premature stop codon was present towards the 3' end of *1Ay-PI428339* coding sequence, which shortened the C-terminal region of the translated subunit (S4 Fig). Interestingly, the central repetitive domain of 1Ay-428339 was longest among the six compared active 1Ay subunits; this explained that the overall length of 1Ay-428339 (608 aa) was comparable to that of other active 1Ay subunits (587–608, S4 Fig), despite the reduction of its C-terminal region. Because of the truncation of C-terminal domain, a conserved cysteine residue normally found in this region of 1Ay was lost in 1Ay-428339, but a new cysteine residue appeared at the end of the repetitive domain in 1Ay-428339 (S4 Fig).

### Investigation of *Glu-A1* locus haplotypes in *T*. *turgidum*

Five *Glu-A1* locus haplotypes (H1, H4, H7, H8 and H9) were identified among 95 *T*. *turgidum* accessions (including two durum wheat lines and ninety-three wild emmer wheat accessions) (Table 1, S2 Table). Compared to the amplicons found in *T*. *urartu*, new alleles were detected for *Xrj5* (719 bp), *Xid3* (null) and *Xrj7* (null) (Table 1). H9 was the most common haplotype (in 48.4% of the accessions, including Langdon, S2 Table), followed by H1 (in 38.9% of the accessions); together, H1 and H9 were found in 87.3% of the *T*. *turgidum* accessions examined (Table 1). H9 differed from H1 in the amplicons of three markers (*Xrj5*, *Xid3* and *Xrj7*), whereas H8, which was the third most frequent haplotype detected (in 9.5% of the accessions), varied from H1 in only the product of *Xrj5* (Table 1). The remaining two haplotypes (H4 and H7) were found in only very small number of accessions (Table 1, S2 Table).

**Fig 3:**
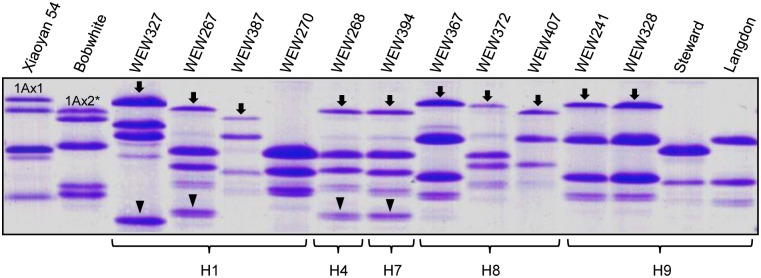
SDS-PAGE detection of HMW-GS composition in *T*. *turgidum Glu-A1* haplotypes. The names of materials were marked on the top of the photo. The protein bands represented the 1Ax subunits were indicated by arrows, and the protein bands represented the 1Ay subunits were indicated by arrowheads. The corresponding haplotypes were marked on the bottom.

The expression of HMW-GSs was investigated in 13 representative *T*. *turgidum* lines carrying H1, H4, H7, H8 or H9. Four lines expressed both 1Ax and 1Ay, while six lines expressed only 1Ax (Fig 3). Neither 1Ax nor 1Ay proteins were detected in three lines (WEW270, Stewart and Langdon, Fig 3). The lines expressing both 1Ax and 1Ay proteins carried *Glu-A1* locus haplotypes H1, H4 or H7, while those lacking both proteins had haplotypes H1 or H9 (Fig 3).

### *Glu-A1* locus haplotypes in common wheat

Five haplotypes (H1, H8, H9, H10 and H11) were identified among 215 common wheat varieties after genotyping with the five *Glu-A1* markers (Table 1, S3 Table). H1 (in 54.4% of the varieties) was the predominant haplotype in the common wheat lines examined, followed by H9 (in 36.7% of the varieties). H10 (in 6.3% of the varieties) and H11 (in 2.3% of the varieties) were new haplotypes found in only common wheat, and the only difference between the two haplotypes was the amplicon size of *Xrj5*, which was 719 bp in H10, but 882 bp in H11 (Table 1). Together, the lines carrying H1 or H9 accounted for 91.2% of the common wheat varieties examined (Table 1).

The *Glu-A1* alleles and composition of HMW-GSs were investigated in the 215 common wheat lines. The *Glu-A1* alleles (i.e., *Glu-A1a*, *-A1b* and *-A1c*) in these lines were readily identified following the standard established previously (Payne and Lawrence 1983) (S3 Table, S5 Fig). Consistent with previous studies (Payne and Lawrence 1983; Shewry et al. 2003), none of the 215 varieties expressed 1Ay, irrespective of their *Glu-A1* allele status; 1Ax1 and 1Ax2* were detected in the varieties with *Glu-A1a* and *-A1b*, respectively; no *Glu-A1* encoded HMW-GSs were detected in the lines having *Glu-A1c* (S3 Table, S5 Fig). The electrophoretic mobility variations of the expressed 1Ax subunits (1Ax1 and 1Ax2*) in common wheat (S5 Fig) were severely reduced compared to those observed in *T*. *urartu* and *T*. *turgidum* (Figs 2 and 3). However, strong correspondence was found between *Glu-A1* allele and *Glu-A1* locus haplotype (S3 Table). The varieties carrying *Glu-A1a* generally had H1, with only one of them belonging to H8; the varieties with *Glu-A1b* had either H10 or H11, whereas those possessing *Glu-A1c* all had H9 (S3 Table).

### Network analysis of *Glu-A1* locus haplotypes

**Fig 4:**
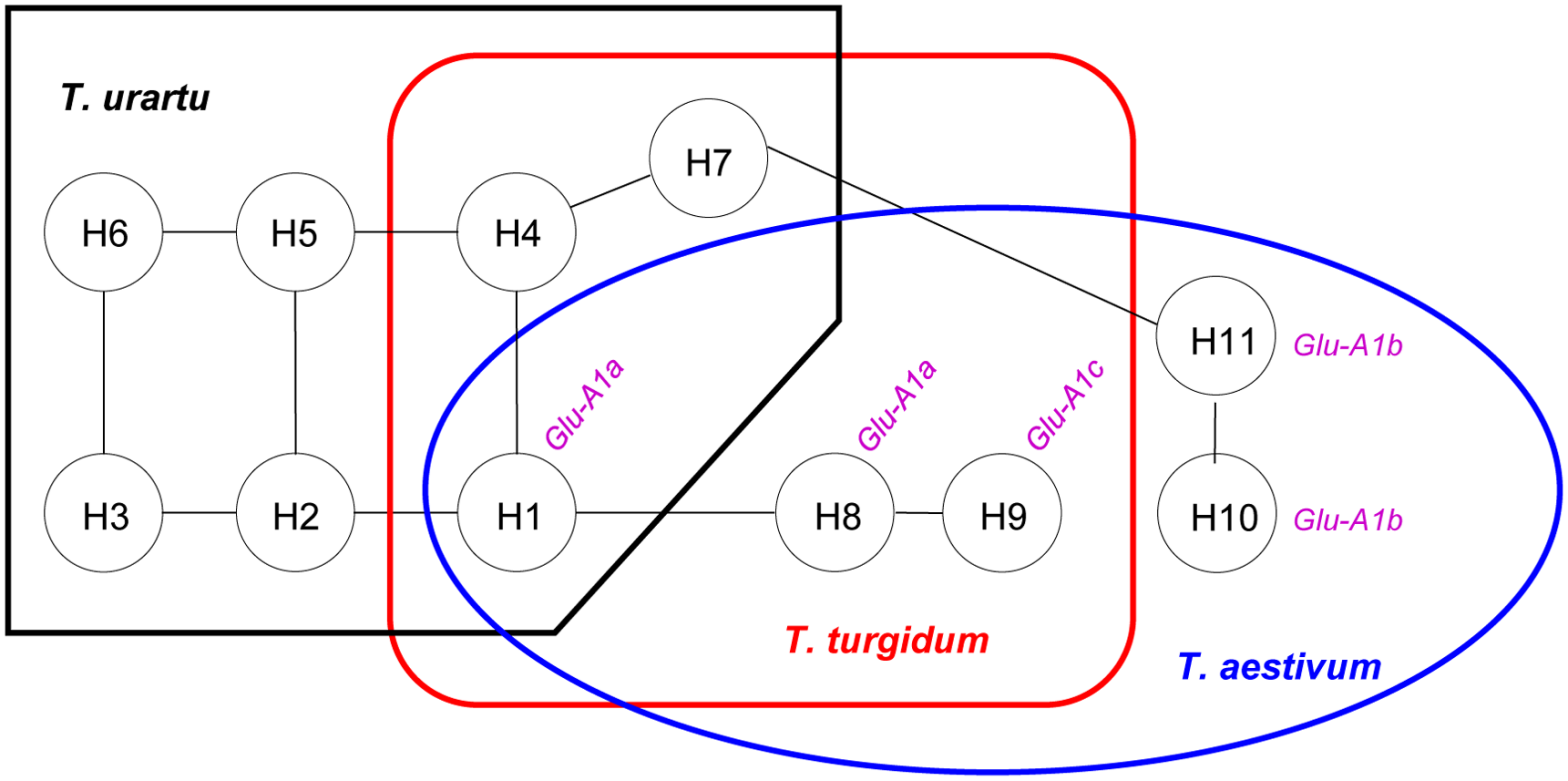
Phylogenetic network analysis of 11 *Glu-A1* haplotypes. The haplotypes detected in *T*. *urartu* were surrounded with black box, those in *T*. *turgidum* were surrounded with red box, and those in common wheat were surrounded with blue circle.

Based on similarities and differences in the alleles of the five DNA markers, phylogenetic network analysis was conducted to investigate possible relationships among the 11 haplotypes. As displayed in Fig 4, complex network connections were found among H1, H2, H3, H4, H5 and H6, but the connections among H1, H8 and H9, as well as those among H7, H10 and H11, were relatively simple. Interestingly, H1, H8, H9, H10 and H11, but except for H7, were all detected in common wheat. The connection among H1, H8 and H9 established the connection between *Glu-A1a* and *Glu-A1c* (Fig 4). The connection between H10 and H11 paralleled with the finding that both haplotypes carried *Glu-A1c* (Fig 4, Table 1).

## Discussion

### Molecular variations of orthologous *Glu-A1* loci in *T*. *urartu*, *T*. *turgidum* and common wheat

Previous knowledge on the sequence variations among orthologous *Glu-A1* loci in *T*. *urartu*, *T*. *turgidum* and common wheat was very limited because it was based on the information from only three genotypes (i.e., G1812, Langdon and Renan) [11–13]. Judging from the genotyping data obtained in this work for multiple *T*. *urartu*, *T*. *turgidum* and common wheat lines, we suggest that the sequence variations of *Glu-A1* locus are much more extensive than previously revealed through BAC sequencing at all three ploidy levels. For example, the generation of three different amplicons by *Xid3* in *T*. *urartu* (655, 718 and 808 bp, Table 1) indicates the presence of at least three *1Ay* alleles in this species. The presence of premature stop codon in these alleles confers additional variations in *1Ay* expression. The positive amplification of *Xrj6* in many *T*. *urartu* accessions (Table 1) indicates the occurrence of *Hawi-2* element in this species although it was not found in the *Glu-A1* locus of G1812 [13]. In *T*. *turgidum*, although the amplification of a 719 bp fragment by *Xrj5* is in agreement with the presence of a truncated *Mite-5* insertion in Langdon (Fig 1A), the production of a 594 bp fragment by the same marker indicates the absence of *Mite-5* insertion in some other accessions of this species (Table 1). The lack of amplification by *Xid3* in many *T*. *turgidum* lines is consistent with the insertion of *Wis-3* element in the *1Ay* gene of Langdon (Fig 1A, Table 1). However, this marker also amplified a 718 bp fragment in *T*. *turgidum* (Table 1), indicating the existence of the *1Ay* gene without *Wis-3* insertion in this species. Similarly, the lack of amplification by *Xrj7* in 48.4% of *T*. *turgidum* accessions is in line with the insertion of *Erika-2* element in Langdon (Fig 1A, Table 1). But this marker also amplified a 961 bp fragment in 51.6% of the *T*. *turgidum* lines (Table 1), indicating that the lack of *Erika-2* insertion is prevalent in *T*. *turgidum*. In common wheat, the amplification of the 882 bp fragment by *Xrj5* is in agreement with the insertion of an intact *Mite-5* element in the receptor kinase b gene of Renan (Fig 1A, Table 1). However, this marker also yielded the 594 and 719 fragments (Table 1), which indicate the lack of *Mite-5* insertion, or the presence of an internally deleted *Mite-5*, in the receptor kinase b gene in common wheat.

The sequence variations of *Glu-A1* in *T*. *turgidum* and common wheat may come mainly from two sources. One source of variations is derived from the ancestral species, and the other is newly evolved. For instance, the 594 bp allele of *Xrj5* is present in all three wheat species examined here, suggesting the transmission of this allele from *T*. *urartu* to *T*. *turgidum* and from *T*. *turgidum* to common wheat. On the other hand, it is likely that the 719 bp and 882 bp alleles of *Xrj5* were formed in *T*. *turgidum* and common wheat, respectively. This may not be surprising as it is now generally accepted that polyploidization promotes the differentiation of new alleles in the evolution of many crop plants including common wheat [43, 44].

Clearly, the use of multiple markers enables rapid assessment of the molecular variations of *Glu-A1* genomic region within and among different wheat species. The rich sequence variations of orthologous *Glu-A1* loci facilitated our haplotype analysis. They may also aid further basic and applied studies of these loci in the future (see below).

### Relationships among *Glu-A1* locus haplotypes in three wheat species

A total of 11 haplotypes were identified for *Glu-A1* locus in the *T*. *urartu*, *T*. *turgidum* and common wheat accessions analyzed in this study (Table 1). Of the seven *Glu-A1* haplotypes of *T*. *urartu*, H1 was shared with *T*. *turgidum* and common wheat, and H4 and H7 were also found in *T*. *turgidum*. These data suggest that *T*. *urartu* haplotypes H1, H4 and H7 may play an important role in shaping the *Glu-A1* haplotypes of *T*. *turgidum* and common wheat. For both *T*. *turgidum* and common wheat, their H1 haplotype was likely derived from *T*. *urartu*. This is consistent with the finding of H1 as a major *Glu-A1* haplotype in all three species (Table 1). Moreover, H1 might also give rise to H8 (Fig 4). *T*. *urartu* haplotype H4 was involved in the differentiation of H7, and H7 contributed to the differentiation of H10 (Fig 4). Because H8 and H9 first appeared in *T*. *turgidum*, they were likely formed in this species, and transmitted to common wheat via hexaploidization. Since H10 and H11 were unique to common wheat, they were probably differentiated at the hexaploid stage. Finally, H2, H3, H5 and H6, detected in only *T*. *urartu* (Table 1, Fig 4), might be more ancient, and contributed to the evolution of H1 and H4.

It is interesting to note that four *T*. *urartu* (H2, H3, H5 and H6) and two *T*. *turgidum* (H4 and H7) *Glu-A1* locus haplotypes were not present in common wheat (Table 1, Fig 4). The six haplotypes might be lost during the hexaploidization process leading to common wheat. The reduction of *Glu-A1* haplotype diversity in common wheat is paralleled with a severe decrease in the expression of 1Ax and 1Ay alleles in this species (Figs 2 and 3, S1 Fig). These reductions may be caused by two main reasons: polyploidy diversity bottlenecks that have been found to act in the evolution of diverse polyploid plants and artificial selections during domestication and modern breeding [45–47].

### Association between *Glu-A1* locus haplotypes and the expression of 1Ax and 1Ay subunits or the three *Glu-A1* alleles of common wheat

In common wheat, 1Ay subunit is always silent, and 1Ax sometimes appear silent [11, 18–20]. Although the expressed 1Ax and 1Ay subunits in some *T*. *urartu* and *T*. *turgidum* accessions have been reported [21–26], expression of 1Ax or 1Ay was unpredictable up to now. In this study, we found strong association between *Glu-A1* locus haplotypes and the expression of 1Ax and 1Ay subunits or the three *Glu-A1* alleles of common wheat. When considering the data from all three species, the association between *Glu-A1* locus haplotypes and the expression of 1Ax and 1Ay subunits appears very complex (S5 Table). For example, among the lines with haplotype H1, some expressed both 1Ax and 1Ay, whereas others specified only 1Ax or none (S5 Table). Nevertheless, within each species, there are some clear associations between specific *Glu-A1* locus haplotypes and subunit expression patterns. In *T*. *urartu*, three main haplotypes showed exclusive (H1 and H4) or predominant (H6) associations with the expression of both 1Ax and 1Ay subunits. In common wheat, H1 and H8 were both associated with the expression of 1Ax1, while H10 and H11 were both linked with 1Ax2* expression; on the other hand, H9 was tightly associated with the silencing of both 1Ax and 1Ay subunits (S5 Table). Because the number of *T*. *turgidum* lines examined for HMW-GS composition in this work was limited, further study is needed to investigate potential associations between specific *Glu-A1* locus haplotypes and subunit expression patterns in this species.

There also exist strong correspondences between *Glu-A1* locus haplotypes and the three *Glu-A1* alleles of common wheat, i.e., H1 and H8 to *Glu-A1a*, H9 to *Glu-A1c*, and H10 and H11 to *Glu-A1b*. We were unable to assess such correspondences for *T*. *urartu* and *T*. *turgidum* because systematic schemes for designating the *Glu-A1* alleles in these two species are still not available. This may be due to the fact that the molecular variations of 1Ax and 1Ay subunits in *T*. *urartu* and *T*. *turgidum* are considerably more complex than those observed in common wheat.

Because of the complexity in *1Ax* and *1Ay* expression in *T*. *urartu* and *T*. *turgidum*, it is difficult to systematically assign alleles to the *Glu-A1* variants in different materials of the two species based on electrophoretic mobility variations of 1Ax and 1Ay subunits. In this situation, *Glu-A1* locus haplotypes, as revealed by this work, may provide a convenient way to describe the *Glu-A1* variants in specific *T*. *urartu* and *T*. *turgidum* genotypes.

### Implications for further research

First, the *Glu-A1* locus haplotypes may be useful for studying the evolutionary origins of the three *Glu-A1* alleles of common wheat. Although *Glu-A1a*, *-A1b* and *-A1c* have been extensively used in contemporary wheat quality breeding programs, their evolutionary origins are still unclear. This is not conducive for mining additional elite *Glu-A1* alleles to aid wheat quality improvement. Based on network relationships of the 11 *Glu-A1* locus haplotypes (Fig 4), and the known evolutionary relationships among *T*. *urartu*, *T*. *turgidum* and common wheat [3, 4], we hypothesize that *Glu-A1a* was originally formed in *T*. *urartu*, and then transmitted to common via two rounds of allopolyploidization. This is supported by the finding of H1 in all three wheat species and the strong association of *Glu-A1a* with H1 in common wheat (Table 1, Fig 4). *Glu-A1b* might also be initially formed in *T*. *urartu*, and then passed to *T*. *turgidum* through tetraploidization and common wheat via hexaploidization. This is supported by 1) the presence of the 158 bp amplicon of *Xid4* diagnostic for *Glu-A1b* in all three wheat species, and 2) the *Glu-A1* locus haplotypes carrying *Glu-A1b* were found in *T*. *urartu* (H7), *T*. *turgidum* (H7) and common wheat (H10 and H11). In contrast to *Glu-A1a* and *-A1b*, *Glu-A1c* might be differentiated in *T*. *turgidum* and transmitted to common wheat through hexaploidization. This is because H9, which carried *Glu-A1c* in common wheat, was also detected in *T*. *turgidum*. Furthermore, we speculate that *Glu-A1c* was probably evolved from *Glu-A1a* since H8, which directly linked with H9, carried *Glu-A1a* (Fig 4). Further studies are needed to verify the above hypothesis and speculation.

Second, of the 11 haplotypes, three (i.e., H1, H4 and H7) are useful for introducing active *1Ay* alleles into common wheat. It is well known that the *1Ay* gene is silenced in common wheat [25, 48], but several studies have shown that introducing diploid or tetraploid wheat *Glu-A1* alleles expressing both 1Ax and 1Ay subunits into common wheat actually conferred improved end-use qualities [22, 23, 27]. Here we found that in *T*. *urartu* the accessions belonged to H1, H4 and H7 all carried the *Glu-A1* alleles with both 1Ax and 1Ay subunits expressed (S1 Table). Notably, in these accessions, the 718 bp allele of *Xid3* was strictly linked with actively expressed *1Ay* alleles (Table 1), including the *1Ay-428339* allele displaying very slow electrophoretic mobility (Fig 2). The 1Ay-428339 subunit, although resembling its allelic counterparts in the primary structure, displays novel variations in the central repetitive domain and C-terminal region (S4 Fig). Notably, its central repetitive domain was considerably longer than that of other active 1Ay alleles (S4 Fig). Because it has been shown that a longer repetitive domain enhances the function of HMW-GSs in gluten and dough functionalities [49, 50], it will be interesting to test the usefulness of 1Ay-428339 for improving wheat end-use quality in the future.

Like in *T*. *urartu*, many of the *T*. *turgidum* accessions with haplotypes H1, H4 or H7 also expressed both 1Ax and 1Ay subunits (Fig 3). Therefore, the active *1Ay* alleles in the *T*. *urartu* and *T*. *turgidum* accessions with haplotypes H1, H4 or H7 may be transferred to common wheat to improve the end-use traits through marker assisted wide hybridization, an approach that has been found valuable in the molecular breeding programs of diverse crops [51, 52].

Finally, based on amplification patterns produced by the five DNA markers (Table 1), we suggest that *Xid4* and *Xrj7* allow efficient detection and distinction of three *Glu-A1* alleles in common wheat. Specifically, *Xid4* alone is sufficient for detecting *Glu-A1b* based on the production of an amplicon of 158 bp. The combined use of *Xid4* and *Xrj7* permits the distinction of *Glu-A1a* from *Glu-A1c*. For *Glu-A1a* the amplicon lengths of the two markers were 176 and 961 bp, respectively, whereas for *Glu-A1c* the two markers yielded a 176 bp amplicon and a null allele, respectively (Table 1). *Xid4* can be considered as a perfect marker [53], because it resides in the coding region of *1Ax* gene (Fig 1A). *Xrj7* is also physically close to *1Ax* and *1Ay* genes (Fig 1A). Thus the use of *Xid4* and *Xrj7* can facilitate efficient and accurate selection of three *Glu-A1* alleles in wheat quality breeding practice.

In summary, the new set of DNA markers developed in this work is useful for studying the molecular variations and evolutionary mechanisms of orthologous *Glu-A1* regions in common wheat and ancestral species. These markers, together with the haplotype variants revealed here, may also aid further research in improving the end-use traits of common wheat.

## Supporting information

S1 Fig Multiple alignment of the DNA sequences of *Xid3* amplicons from Renan, Langdon, G1812 and PI428204, as well as Renan *1By* and *1Dy*. (PPTX)

S2 Fig Multiple alignment of the DNA sequences of *Xid4* amplicons from Renan, Langdon and G1812, as well as Renan *1Bx* and *1Dx*. (PPTX)

S3 Fig Multiple alignment of the DNA sequences of *Xrj5* amplicons from G1812, Langdon and Renan. (PPTX)

S4 Fig Comparison of the deduced amino acid sequences of *1Ay* genes from *T*. *urartu*. (PPTX)

S5 Fig SDS-PAGE detection of HMW-GS composition in common wheat *Glu-A1* haplotypes. (PPTX)

S1 Table *Glu-A1* locus haplotypes detected in *T*. *urartu* accessions. (XLSX)

S2 Table *Glu-A1* locus haplotypes detected in *T*. *turgidum* accessions. (XLSX)

S3 Table Haplotype variation of *Glu-A1* locus in the common wheat varieties. (XLSX)

S4 Table The primers used in this study. (DOCX)

S5 Table Summary of the expression of 1Ax and 1Ay subunits in different wheat species. (DOCX)
