# Supplementary material for: Development of a new set of molecular markers for examining Glu-A1 variants in common wheat and ancestral species
Source: PLoS One. 2017 Jul 6;12(7):e0180766. doi: 10.1371/journal.pone.0180766 (PMC5500356; doi:10.1371/journal.pone.0180766)
Supplement: S1 Fig — (PPTX) [file pone.0180766.s001.pptx]

## Slide 1
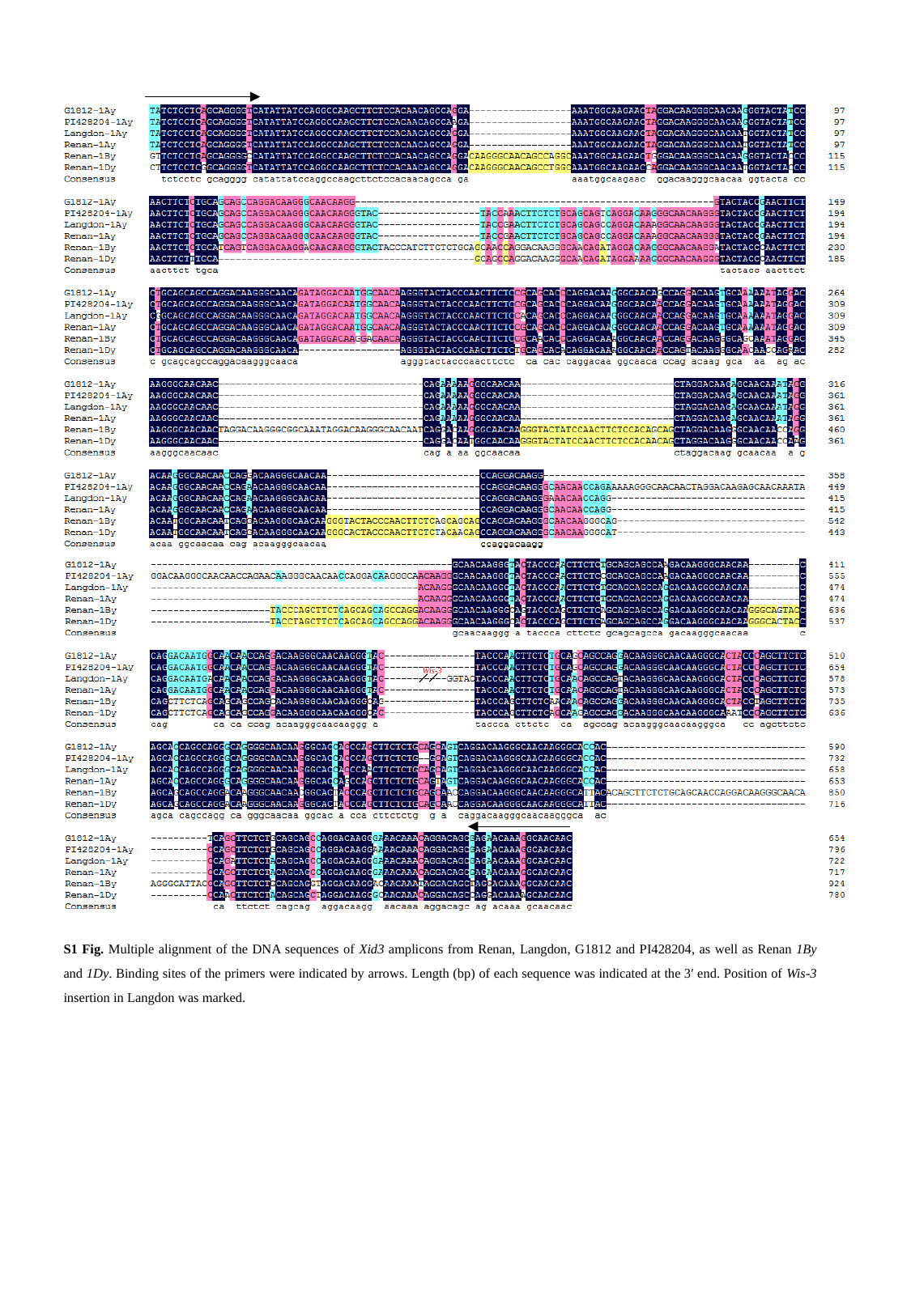

Wis-3
S1 Fig. Multiple alignment of the DNA sequences of Xid3 amplicons from Renan, Langdon, G1812 and PI428204, as well as Renan 1By and 1Dy. Binding sites of the primers were indicated by arrows. Length (bp) of each sequence was indicated at the 3′ end. Position of Wis-3 insertion in Langdon was marked.
