# Supplementary material for: Development of a new set of molecular markers for examining Glu-A1 variants in common wheat and ancestral species
Source: PLoS One. 2017 Jul 6;12(7):e0180766. doi: 10.1371/journal.pone.0180766 (PMC5500356; doi:10.1371/journal.pone.0180766)
Supplement: S2 Fig — (PPTX) [file pone.0180766.s002.pptx]

## Slide 1
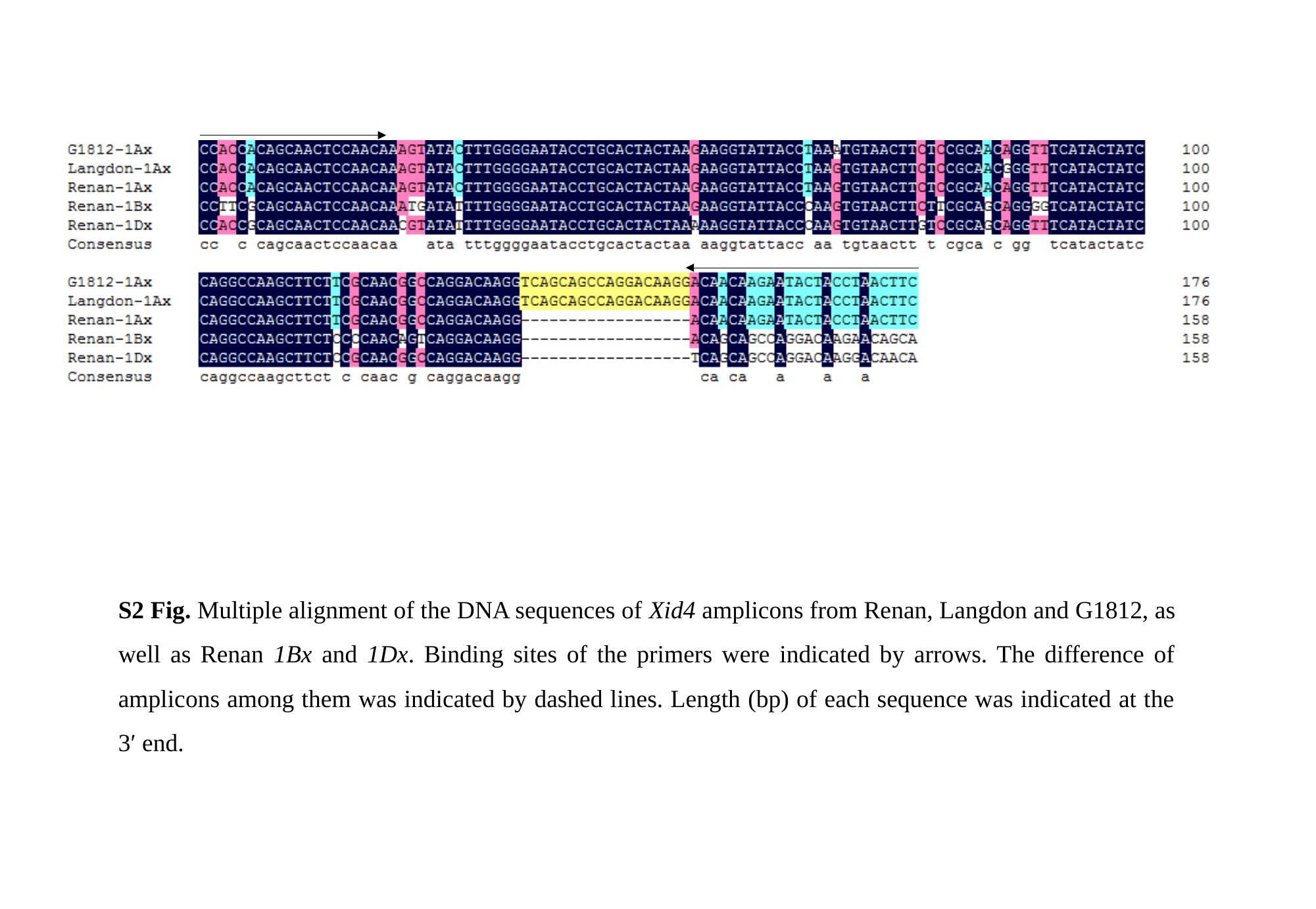

S2 Fig. Multiple alignment of the DNA sequences of Xid4 amplicons from Renan, Langdon and G1812, as well as Renan 1Bx and 1Dx. Binding sites of the primers were indicated by arrows. The difference of amplicons among them was indicated by dashed lines. Length (bp) of each sequence was indicated at the 3′ end.
