# Supplementary material for: Development of a new set of molecular markers for examining Glu-A1 variants in common wheat and ancestral species
Source: PLoS One. 2017 Jul 6;12(7):e0180766. doi: 10.1371/journal.pone.0180766 (PMC5500356; doi:10.1371/journal.pone.0180766)
Supplement: S3 Fig — (PPTX) [file pone.0180766.s003.pptx]

## Slide 1
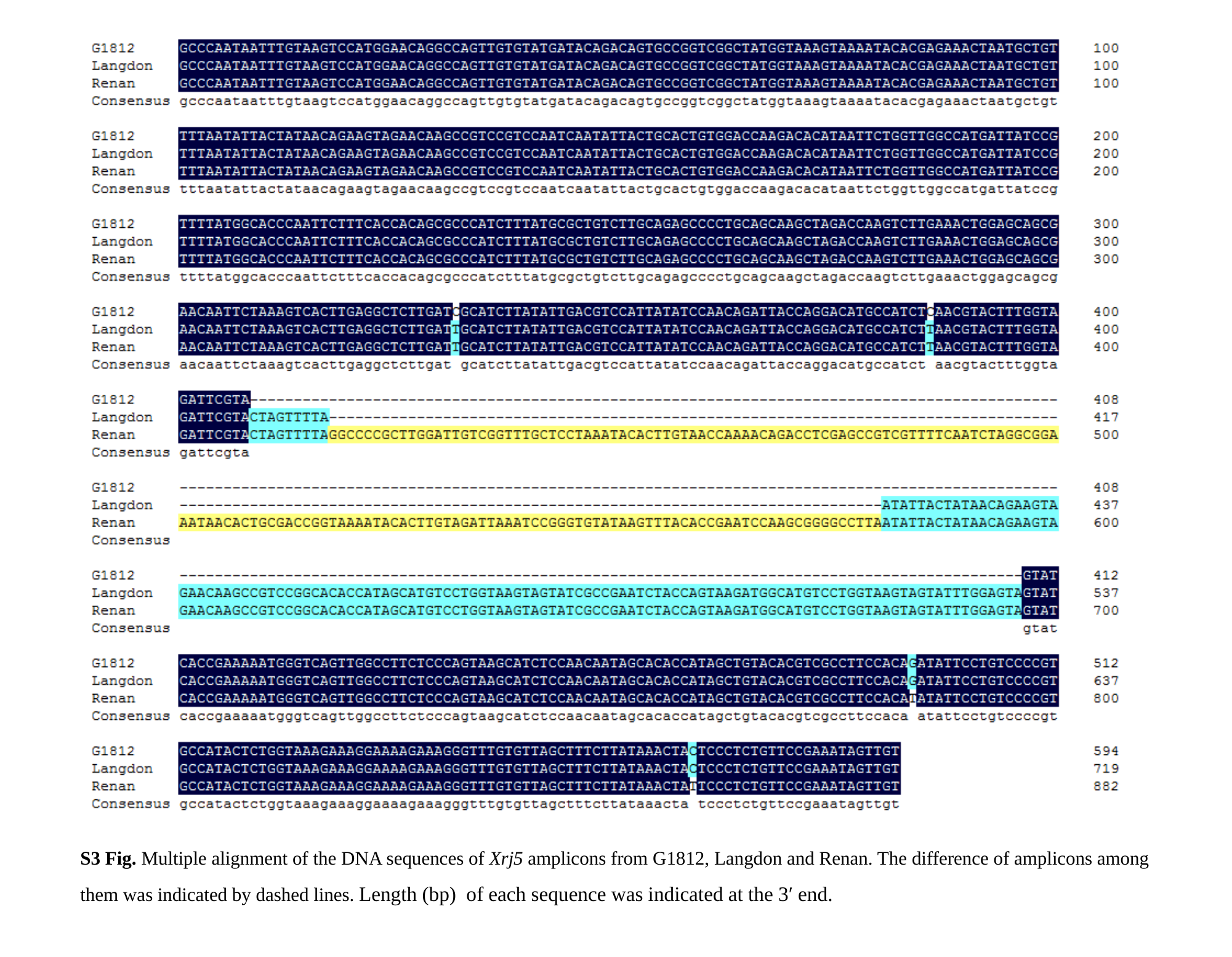

S3 Fig. Multiple alignment of the DNA sequences of Xrj5 amplicons from G1812, Langdon and Renan. The difference of amplicons among them was indicated by dashed lines. Length (bp) of each sequence was indicated at the 3′ end.
