# Supplementary material for: Development of a new set of molecular markers for examining Glu-A1 variants in common wheat and ancestral species
Source: PLoS One. 2017 Jul 6;12(7):e0180766. doi: 10.1371/journal.pone.0180766 (PMC5500356; doi:10.1371/journal.pone.0180766)
Supplement: S4 Fig — (PPTX) [file pone.0180766.s004.pptx]

## Slide 1
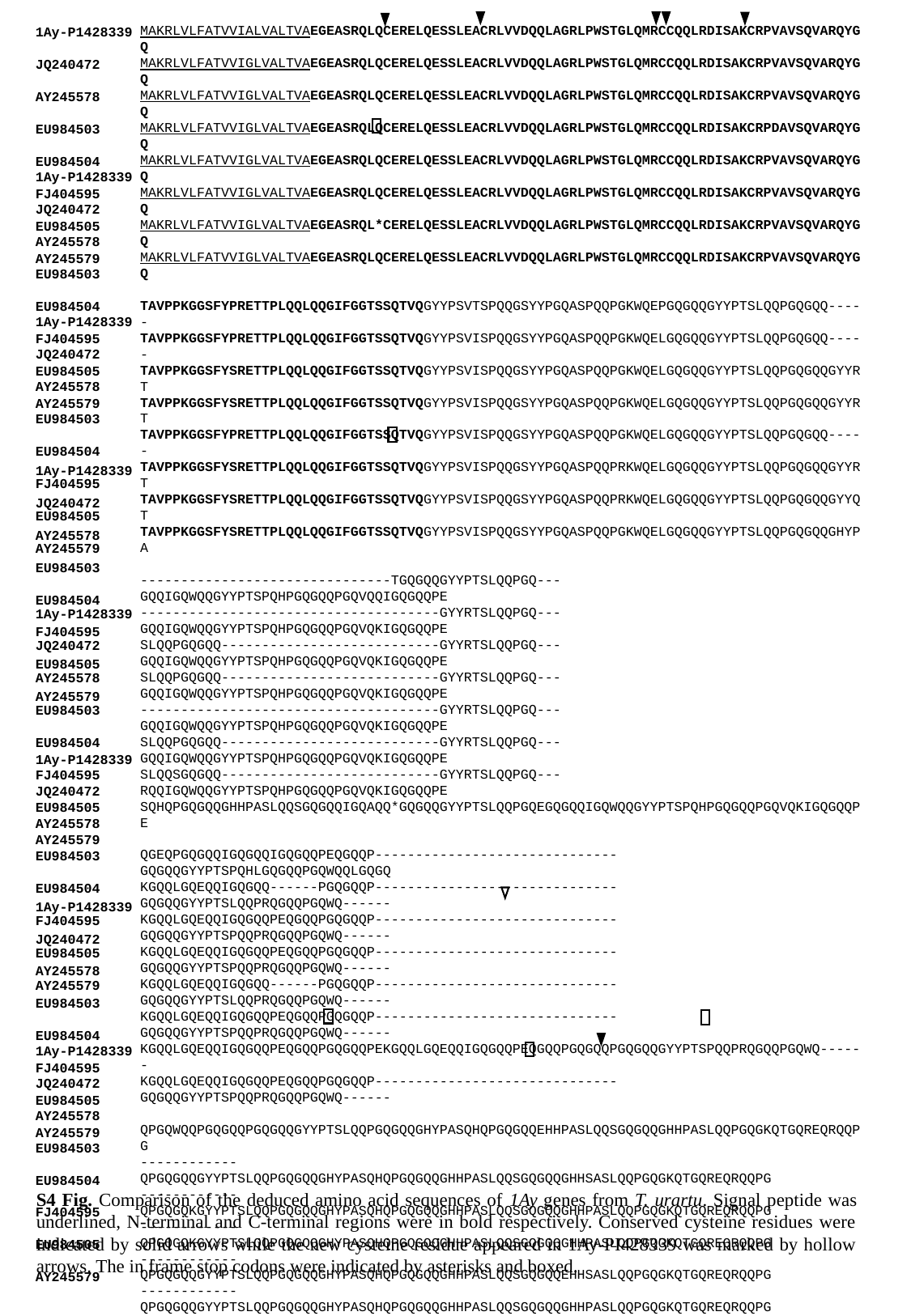

MAKRLVLFATVVIALVALTVAEGEASRQLQCERELQESSLEACRLVVDQQLAGRLPWSTGLQMRCCQQLRDISAKCRPVAVSQVARQYGQ
MAKRLVLFATVVIGLVALTVAEGEASRQLQCERELQESSLEACRLVVDQQLAGRLPWSTGLQMRCCQQLRDISAKCRPVAVSQVARQYGQ
MAKRLVLFATVVIGLVALTVAEGEASRQLQCERELQESSLEACRLVVDQQLAGRLPWSTGLQMRCCQQLRDISAKCRPVAVSQVARQYGQ
MAKRLVLFATVVIGLVALTVAEGEASRQLQCERELQESSLEACRLVVDQQLAGRLPWSTGLQMRCCQQLRDISAKCRPDAVSQVARQYGQ
MAKRLVLFATVVIGLVALTVAEGEASRQLQCERELQESSLEACRLVVDQQLAGRLPWSTGLQMRCCQQLRDISAKCRPVAVSQVARQYGQ
MAKRLVLFATVVIGLVALTVAEGEASRQLQCERELQESSLEACRLVVDQQLAGRLPWSTGLQMRCCQQLRDISAKCRPVAVSQVARQYGQ
MAKRLVLFATVVIGLVALTVAEGEASRQL*CERELQESSLEACRLVVDQQLAGRLPWSTGLQMRCCQQLRDISAKCRPVAVSQVARQYGQ
MAKRLVLFATVVIGLVALTVAEGEASRQLQCERELQESSLEACRLVVDQQLAGRLPWSTGLQMRCCQQLRDISAKCRPVAVSQVARQYGQ
TAVPPKGGSFYPRETTPLQQLQQGIFGGTSSQTVQGYYPSVTSPQQGSYYPGQASPQQPGKWQEPGQGQQGYYPTSLQQPGQGQQ-----
TAVPPKGGSFYPRETTPLQQLQQGIFGGTSSQTVQGYYPSVISPQQGSYYPGQASPQQPGKWQELGQGQQGYYPTSLQQPGQGQQ-----
TAVPPKGGSFYSRETTPLQQLQQGIFGGTSSQTVQGYYPSVISPQQGSYYPGQASPQQPGKWQELGQGQQGYYPTSLQQPGQGQQGYYRT
TAVPPKGGSFYSRETTPLQQLQQGIFGGTSSQTVQGYYPSVISPQQGSYYPGQASPQQPGKWQELGQGQQGYYPTSLQQPGQGQQGYYRT
TAVPPKGGSFYPRETTPLQQLQQGIFGGTSSQTVQGYYPSVISPQQGSYYPGQASPQQPGKWQELGQGQQGYYPTSLQQPGQGQQ-----
TAVPPKGGSFYSRETTPLQQLQQGIFGGTSSQTVQGYYPSVISPQQGSYYPGQASPQQPRKWQELGQGQQGYYPTSLQQPGQGQQGYYRT
TAVPPKGGSFYSRETTPLQQLQQGIFGGTSSQTVQGYYPSVISPQQGSYYPGQASPQQPRKWQELGQGQQGYYPTSLQQPGQGQQGYYQT
TAVPPKGGSFYSRETTPLQQLQQGIFGGTSSQTVQGYYPSVISPQQGSYYPGQASPQQPGKWQELGQGQQGYYPTSLQQPGQGQQGHYPA
-------------------------------TGQGQQGYYPTSLQQPGQ---GQQIGQWQQGYYPTSPQHPGQGQQPGQVQQIGQGQQPE
-------------------------------------GYYRTSLQQPGQ---GQQIGQWQQGYYPTSPQHPGQGQQPGQVQKIGQGQQPE
SLQQPGQGQQ---------------------------GYYRTSLQQPGQ---GQQIGQWQQGYYPTSPQHPGQGQQPGQVQKIGQGQQPE
SLQQPGQGQQ---------------------------GYYRTSLQQPGQ---GQQIGQWQQGYYPTSPQHPGQGQQPGQVQKIGQGQQPE
-------------------------------------GYYRTSLQQPGQ---GQQIGQWQQGYYPTSPQHPGQGQQPGQVQKIGQGQQPE
SLQQPGQGQQ---------------------------GYYRTSLQQPGQ---GQQIGQWQQGYYPTSPQHPGQGQQPGQVQKIGQGQQPE
SLQQSGQGQQ---------------------------GYYRTSLQQPGQ---RQQIGQWQQGYYPTSPQHPGQGQQPGQVQKIGQGQQPE
SQHQPGQGQQGHHPASLQQSGQGQQIGQAQQ*GQGQQGYYPTSLQQPGQEGQGQQIGQWQQGYYPTSPQHPGQGQQPGQVQKIGQGQQPE
QGEQPGQGQQIGQGQQIGQGQQPEQGQQP------------------------------GQGQQGYYPTSPQHLGQGQQPGQWQQLGQGQ
KGQQLGQEQQIGQGQQ------PGQGQQP------------------------------GQGQQGYYPTSLQQPRQGQQPGQWQ------
KGQQLGQEQQIGQGQQPEQGQQPGQGQQP------------------------------GQGQQGYYPTSPQQPRQGQQPGQWQ------
KGQQLGQEQQIGQGQQPEQGQQPGQGQQP------------------------------GQGQQGYYPTSPQQPRQGQQPGQWQ------
KGQQLGQEQQIGQGQQ------PGQGQQP------------------------------GQGQQGYYPTSLQQPRQGQQPGQWQ------
KGQQLGQEQQIGQGQQPEQGQQPGQGQQP------------------------------GQGQQGYYPTSPQQPRQGQQPGQWQ------
KGQQLGQEQQIGQGQQPEQGQQPGQGQQPEKGQQLGQEQQIGQGQQPEQGQQPGQGQQPGQGQQGYYPTSPQQPRQGQQPGQWQ------
KGQQLGQEQQIGQGQQPEQGQQPGQGQQP------------------------------GQGQQGYYPTSPQQPRQGQQPGQWQ------
QPGQWQQPGQGQQPGQGQQGYYPTSLQQPGQGQQGHYPASQHQPGQGQQEHHPASLQQSGQGQQGHHPASLQQPGQGKQTGQREQRQQPG
------------QPGQGQQGYYPTSLQQPGQGQQGHYPASQHQPGQGQQGHHPASLQQSGQGQQGHHSASLQQPGQGKQTGQREQRQQPG
------------QPGQGQKGYYPTSLQQPGQGQQGHYPASQHQPGQGQQGHHPASLQQSGQGQQGHHPASLQQPGQGKQTGQREQRQQPG
------------QPGQGQKGYYPTSLQQPGQGQQGHYPASQHQPGQGQQGHHPASLQQSGQGQQGHHPASLQQPGQGKQTGQREQRQQPG
------------QPGQGQQGYYPTSLQQPGQGQQGHYPASQHQPGQGQQGHHPASLQQSGQGQQEHHSASLQQPGQGKQTGQREQRQQPG
------------QPGQGQQGYYPTSLQQPGQGQQGHYPASQHQPGQGQQGHHPASLQQSGQGQQGHHPASLQQPGQGKQTGQREQRQQPG
------------QPGQGQQGYYPTSLQQPGQGQQGHYPASQHQPGQGQQGHHPASLQQSGQGQQGHHPASLQQPGQGKQTGQREQRQQPG
------------QPGQGQKGYYPTSLQQPGQGQQGHYPASQHQPGQGQQGHHPASLQQSGQGQQGHHPASLQQPGQGKQTGQREQRQQPG
QGQQTGQGQQPEQEQQPGQGQQGYYPTSPQQPGQGQQPEQWEQLGQGQQGHYPASLQQPGQGQQGHYPASLQQPGQGQPGQTQQPGQGQP
QGQQTGQGQQPEQEQQPGQGQQGYYPTYLQQPGQGQQPEQWQQLGQGQQGHYPASLQQSGQGQQGHYPASLQQPGQGQPGQTQQPGQGQ-
QGQQTGQGQQPEQEQQPGQGRQGYYPTYPQQPGQGQQPEQWQQPGQGQQRHYPASLQQSGQGQQGHYPASLQQPGQGQPGQTQQPGQGQ-
QGQQTGQGQQPEQEQQPGQGQQGYYPTYPQQPGQGQQPEQWQQPGQGQQRHYPASLQQSGQGQQGHYPASLQQPGQGQPGQTQQPGQGQ-
QGQQTGQGQQPEQEQQPGQGQQGYYPTYLQQPGQGQQPEQWQQLGQGQQGHYPASLQQSGQGQQGHYPASLQQPGQGQPGQTQQPGQGQ-
QGQQTGQGQQPEQEQQPGQGQQGYYPTYSQQPGQGQQPEQWQQPGQGQQRHYPASLQQSGQGQQGHYPTSLQQLGQGQPGQTQQPGQGQ-
QGQQTGQGQQPEQEQQPGQGQQGYYPTYPQQPGQGQQPEQWQQPGQGQQRHYPASLQQSGQGQQGHYPTSLQQPGQGQPGQTQQPGQGQ-
QGQQTGQGQQPEQEQQPGQGRQGYYPTYPQQSGQGQQPEQWQQPGQGQQRHYPASLQQSGQGQQGHYPASLQQPGQGQPGQTQQPGQGQ-
GQTQQPGQGQPGETQQSGQGQQGYYPTSPQQPGQGQQPGQGQQGHCPTSPQQPGQAQQPGQGQQTGQVQQLGQGQQGYYPTSLQQPGQEQ
---------HPEQEEQPGQGQQGYYPTSPQQPGQGQQPGQGQQGHFPTS----GQAQQPGQGQQIGQAQQLGQGQQGYYPTSLQQPGQEQ
---------HPEQEEQPGQGQQGYYPTSPQQPGQGQQPGQGQQGHFPTF----GQAQQPGQGQQIGQAQQQGQGQQGYYPTSLQQPGQEQ
---------HPEQEEQPGQGQQGYYPTSPQQPGQGQQPGQGQQGHFPTS----GQAQQPGQGQQIGQAQQLGQGQQGYYPTSLQQPGQEQ
---------HPEQEEQPGQGQQGYYPTSPQQPGQGQQPGQGQQGHFPTS----GQAQQPGQGQQIGQAQQLGQGQQGYYPTSLQQPGQEQ
---------HPEQEEQPGQGQQGYYPTSPQQPGQGQQPGQGQQGHFPTS----GQAQQPGQGQQIGQAQQLGQGQQGYYPTSLQQPGQEQ
---------HPEQEEQPGQGQQGYYPTSPQQPGQGQQPGQGQQGHFPTS----GQAQQPGQGQQIGHAQQLGQGQQGYYPTSLQQPGQEQ
---------HPEQEEQPGQGQQG*YPTSPQQPGQGQQPGQGQQGHFPTF----GQAQQPGQGQQIGQAQQ*GQGQQGYYPTSLQQPGQEQ
QSGQGQQLGQGHQPEQGQQSGQEQQGYDNPYHVSVEQQVASPKVAKAH*PTAQLPTMCQMEGGDALSASQ 630
QSGQGQQLGQGHQPGQGQQSGQEQQGYDSPYHVSVEQQAASPKVAKAHHPVAQLPTMCQMEGGDTLSASQ 587
QSGQGQQLGQGHQPGQGQQSGQEQQGYDSPYHVSVEQQAASPKVAKAHHPVAQLPTMCQMEGGDALSASQ 608
QSGQGQQLGQGHQPGQGQQSGQEQQGYDSPYHVSVEQQAASPKVAKAHHPVAQLPTMCQMEGGDALSASQ 608
QSGQGQQLGQGHQPGQGQQSGQEQQGYDSPYHVSVEQQAASPKVAKAHHPVAQLPTMCQMEGGDALSASQ 587
QSGQGQQLGQGHQPGQGQQSGQEQQGYDSPYHVSVEQQAASPKVAKAHHPVAQLPTMCQMEGGDALSASQ 608
QSGQGQQLGQGHQPGQGQQSGQEQQGYDSPYHVSVEQQAASPKVAKAHHPVAQLPTMCQMEGGDALSASQ 637
QSGQGQQLGQGHQPGQGQQSGQEQQGYDSPYHVSVEQQAASPKVAKAHHPVAQLPTMCQMEGGDALSASQ 635
1Ay-P1428339
JQ240472
AY245578
EU984503
EU984504
FJ404595
EU984505
AY245579
1Ay-P1428339
JQ240472
AY245578
EU984503
EU984504
FJ404595
EU984505
AY245579
1Ay-P1428339
JQ240472
AY245578
EU984503
EU984504
FJ404595
EU984505
AY245579
1Ay-P1428339
JQ240472
AY245578
EU984503
EU984504
FJ404595
EU984505
AY245579
1Ay-P1428339
JQ240472
AY245578
EU984503
EU984504
FJ404595
EU984505
AY245579
1Ay-P1428339
JQ240472
AY245578
EU984503
EU984504
FJ404595
EU984505
AY245579
1Ay-P1428339
JQ240472
AY245578
EU984503
EU984504
FJ404595
EU984505
AY245579
1Ay-P1428339
JQ240472
AY245578
EU984503
EU984504
FJ404595
EU984505
AY245579
S4 Fig. Comparison of the deduced amino acid sequences of 1Ay genes from T. urartu. Signal peptide was underlined, N-terminal and C-terminal regions were in bold respectively. Conserved cysteine residues were indicated by solid arrows while the new cysteine residue appeared in 1Ay-PI428339 was marked by hollow arrows. The in frame stop codons were indicated by asterisks and boxed.
