# Supplementary material for: Development of a new set of molecular markers for examining Glu-A1 variants in common wheat and ancestral species
Source: PLoS One. 2017 Jul 6;12(7):e0180766. doi: 10.1371/journal.pone.0180766 (PMC5500356; doi:10.1371/journal.pone.0180766)
Supplement: S5 Fig — (PPTX) [file pone.0180766.s005.pptx]

## Slide 1
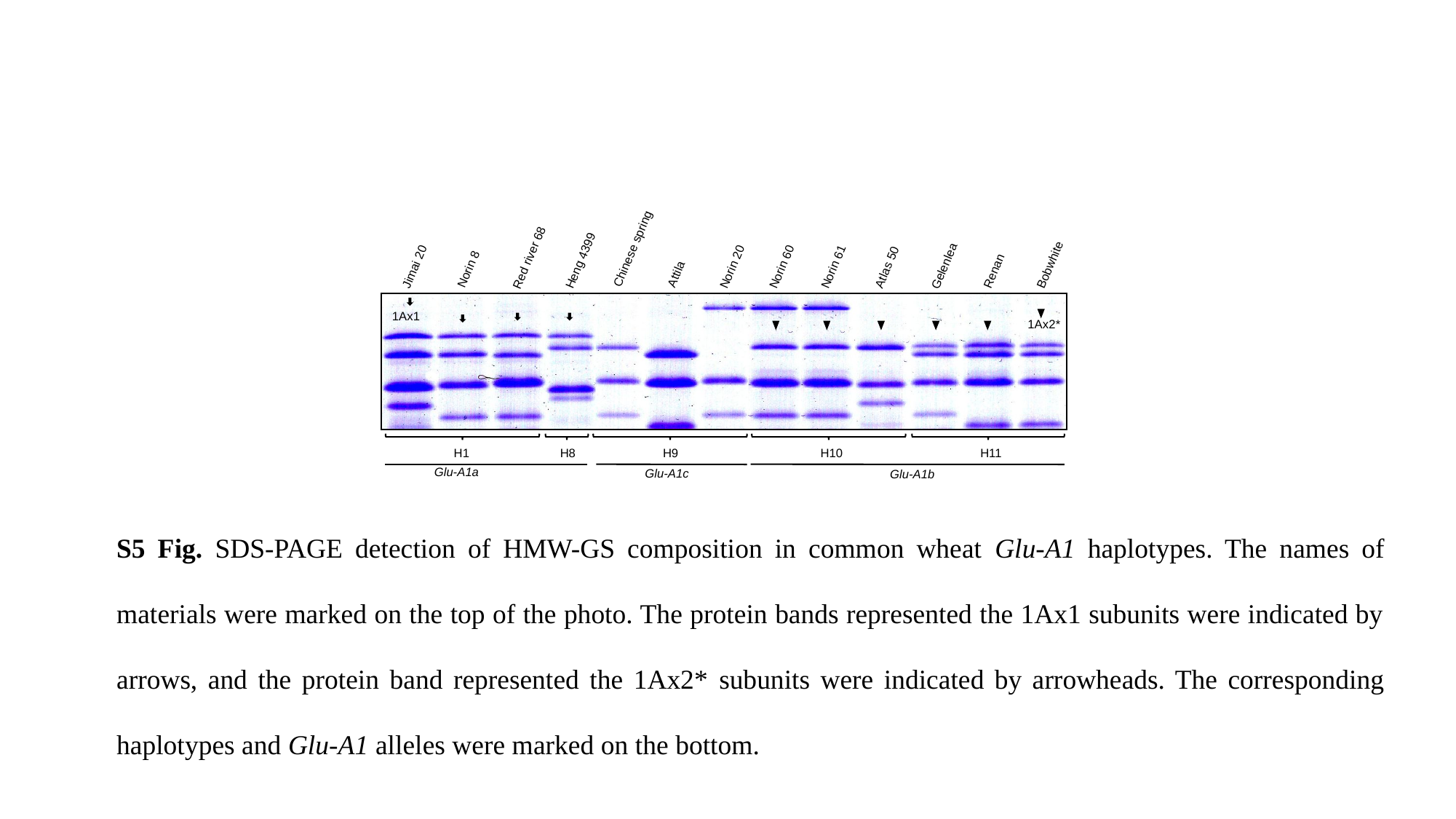

Chinese spring
Red river 68
Heng 4399
Bobwhite
Gelenlea
Jimai 20
Norin 20
Norin 60
Norin 61
Atlas 50
Norin 8
Renan
Attila
1Ax1
1Ax2*
H1
H8
H10
H11
H9
Glu-A1a
Glu-A1c
Glu-A1b
S5 Fig. SDS-PAGE detection of HMW-GS composition in common wheat Glu-A1 haplotypes. The names of materials were marked on the top of the photo. The protein bands represented the 1Ax1 subunits were indicated by arrows, and the protein band represented the 1Ax2* subunits were indicated by arrowheads. The corresponding haplotypes and Glu-A1 alleles were marked on the bottom.
