# Supplementary material for: Development of a new set of molecular markers for examining Glu-A1 variants in common wheat and ancestral species
Source: PLoS One. 2017 Jul 6;12(7):e0180766. doi: 10.1371/journal.pone.0180766 (PMC5500356; doi:10.1371/journal.pone.0180766)
Supplement: S4 Table — (DOCX) [file pone.0180766.s009.docx]

**S4 Table.** **The primers used in this study**

| Name | Sequence (5′ - 3 ′) | Amplification | Anticipated amplicon size (bp) |
| --- | --- | --- | --- |
| Xid3-F | TATCTCCTCAGCAGGGGTCA | *Xid3* | 655, 718 or 808 |
| Xid3-R | GGTTGTTGCCTTTGTTCTC |  |  |
| Xid4-F | CCACCACAGCAACTCCAACA | *Xid4* | 158 or 176 |
| Xid4-R | GAAGTTAGGTAGTATTCTTGTTGTC |  |  |
| Xrj5-F | GCCCAATAATTTGTAAGTCCA | *Xrj5* | 594, 719 or 882 |
| Xrj5-R | ACAACTATTTCGGAACAGAGG |  |  |
| Xrj6-F | CGACGGCACTTCCGATTAT | *Xrj6* | 797 or Null |
| Xrj6-R | CTCACGGTTGCTTTCTCCC |  |  |
| Xrj7-F | GGTTGGATCGTGAAGACGTT | *Xrj7* | 961 or Null |
| Xrj7-R | GGTCGCTACAAATTCATCTATCT |  |  |
