# Supplementary material for: Development of a new set of molecular markers for examining Glu-A1 variants in common wheat and ancestral species
Source: PLoS One. 2017 Jul 6;12(7):e0180766. doi: 10.1371/journal.pone.0180766 (PMC5500356; doi:10.1371/journal.pone.0180766)
Supplement: S5 Table — (DOCX) [file pone.0180766.s010.docx]

**S5 Table. Summary of the expression of 1Ax and 1Ay subunits in 11 *Glu-A1* locus haplotypes in three wheat species**

| Species | *Glu-A1* locus haplotypes | | | | | | | | | | |
| --- | --- | --- | --- | --- | --- | --- | --- | --- | --- | --- | --- |
|  | H1 | H2 | H3 | H4 | H5 | H6 | H7 | H8 | H9 | H10 | H11 |
| *T. urartu* | 1Ax + 1Ay (30)^a^ | 1Ax  (2) | 1Ax (1) | 1Ax + 1Ay (27) | 1Ax + 1Ay (3)  1Ax  (17) | 1Ax + 1Ay (15)  1Ax  (3) | 1Ax + 1Ay (1) | --- | --- | --- | --- |
| *T. turgidum* | 1Ax + 1Ay (2)  1Ax  (1)  Null  (1) | --- | --- | 1Ax + 1Ay (1) | --- | --- | 1Ax + 1Ay (1) | 1Ax  (3) | 1Ax  (2)  Null  (2) | --- | --- |
| *T. aestivum* | 1Ax1  (117) | --- | --- | --- | --- | --- | --- | 1Ax1  (1) | Null  (79) | 1Ax2* (13) | 1Ax2* (5) |

^a^ The value in the brackets indicates the number of accessions expressing both subunits (1Ax + 1Ay), one subunit (1Ax), or none of the two subunits (null).
